# Ultrasound-Assisted Extraction of Inorganic Elements and Antioxidants from Gingerbread Cookies Using Natural Deep Eutectic Solvents

**DOI:** 10.3390/foods14183165

**Published:** 2025-09-11

**Authors:** Agnieszka Kowaluk, Jean Guédon, Natalia Kryska, Dobrochna Rabiej-Kozioł, Michał Strzelec, Aleksandra Szydłowska-Czerniak

**Affiliations:** 1 Department of Analytical Chemistry and Applied Spectroscopy, Faculty of Chemistry, Nicolaus Copernicus University in Toruń, 87-100 Toruń, Poland; 503708@doktorant.umk.pl (A.K.); 302274@stud.umk.pl (N.K.); d.rabiej@umk.pl (D.R.-K.); 2 Central Office of Measures, Laboratory of Electrochemical and Inorganic Analyzes, Department of Physical and Environmental Chemistry, 00-139 Warszawa, Poland; michal.strzelec@gum.gov.pl; 3 Institut Universitaire de Technologie (IUT) de Rennes, Universite de Rennes, F-35000 Rennes, France; jean.guedon@etudiant.univ-rennes.fr

**Keywords:** confectionery products, green extraction, inorganic analytes, antioxidant capacity, inductively coupled plasma mass spectrometry, spectrophotometry

## Abstract

In the present study, ultrasound-assisted extraction using deep eutectic solvents was proposed for the preparation of uniced and iced gingerbread cookies prior to the determination of four macronutrients (potassium, sodium, magnesium, calcium), four micronutrients (manganese, zinc, iron, copper), the presence of toxic metal (cadmium), and antioxidant capacity. With the addition of 30% water in each green solvent, three acidic deep eutectic solvents, comprising xylitol with malic acid, choline chloride with malic acid, and choline chloride with lactic acid, were tested for their efficiencies in the simultaneous extraction of elements and antioxidants. The synthesized deep eutectic solvents were characterized by infrared spectroscopy, which provided evidence of generating new hydrogen bonds between two components of these solvents. Element profiles were analyzed by inductively coupled plasma–mass spectrometry after the extraction using green solvents and the microwave-assisted acid digestion of gingerbread samples. It was found that two deep eutectic solvents containing malic acid exhibited high abilities for solubilization of macronutrients and manganese from the samples studied, while the best extraction efficiencies for Zn, Fe and Cu micronutrients were achieved when the lactic acid-based deep eutectic solvent was used. However, the antioxidant capacity, evaluated by 2,2′-azino-bis(3-ethylbenzothiazoline-6-sulfonic acid) (ABTS), 2,2-diphenyl-1-picrylhydrazyl (DPPH), and cupric reducing antioxidant capacity (CUPRAC) methods, led to the selection of choline chloride–lactic acid as the most promising green solvent for extracting antioxidants from two types of gingerbread cookies. The deep eutectic solvent-based extraction conforms to the principles of green chemistry and is suitable for releasing elements and antioxidants from gingerbread cookies.

## 1. Introduction

Europe is the largest consumer of gingerbread products, holding 30% of the market share in a market stranglehold. The European Union countries with the highest gingerbread production were Germany, Spain, and the Netherlands, which comprise 60% of the total output, while Poland, Belgium, Greece, Sweden, Portugal, Hungary, and Italy account for a further 31% [1]. Polish gingerbread, especially that produced in Toruń since the Middle Ages, has become an ambassador of culinary culture on the global stage. Generally, gingerbread refers to sweet breads, cakes, or cookies made with wheat flour, sugar, spices such as ginger, cinnamon, cloves, nutmeg, chemical leaveners, fat, eggs, and honey.

**Figure 1:**
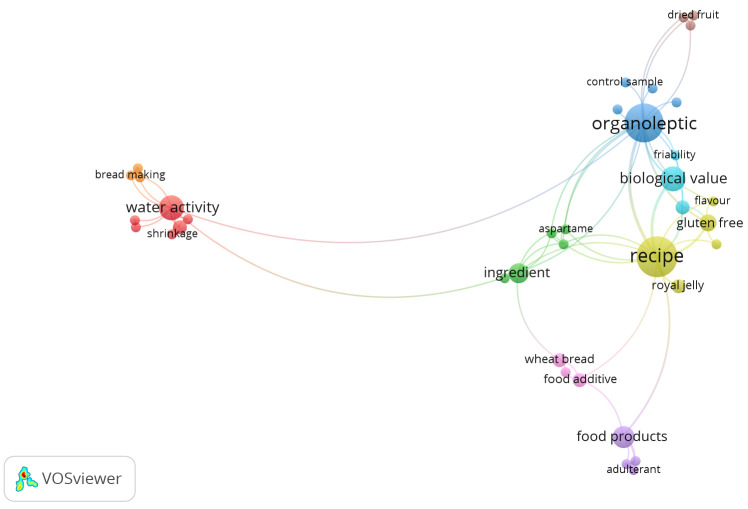
The co-occurrence map of keywords indicating the publication trends on gingerbread products.

A two-dimensional map depicting co-occurrence networks based on term frequency and the examination of the 96 keywords obtained from the scientific papers that provide insight into gingerbread products was generated using VOSviewer (version 1.6.20) software (Figure 1). The VOSviewer analysis of articles on gingerbread products revealed nine distinct clusters, highlighting current trends in research based on gingerbread processing, their ingredients, sensory analysis, and physicochemical and nutritional properties.

It is known that in large-scale gingerbread production, honey can be replaced by other sweeteners, including sucrose hydrolysate, treacle or molasses [2]. Moreover, in the technological processes, gingerbread formulations were improved by using more nutritionally valuable flours, such as rye [3,4,5], quinoa and defatted apple seeds [6], defatted sunflower seed kernels [7], mama-cadela seeds [8], and sea buckthorn [9] richer in dietary fibers, vitamins, minerals, and phenolic compounds than wheat flour. The increment of antioxidant properties and sensory quality of gingerbread cakes was also achieved by the addition of 25% pumpkin, tomato, and beetroot purée [10]. Additionally, functional ingredients, including powder and extracts from rose hip pulp as natural dyes and jam from carrot roots, enhanced the nutritional, antioxidant, and biological values of the gingerbread cookies [3,11]. In our previous paper, the increase in phenolic compounds and the antioxidant properties of gingerbread cookies was caused by their covering with chocolate fortified by elderflower dry extract and filling with elderberry juice concentrate [12]. Meanwhile, the replacement of cocoa powder in coating with grape seed powder from grape pomace, known as a residue of winemaking, improved sensory attributes and shelf life of gingerbread [13]. Furthermore, food waste, such as powder from chicken eggshells, rich in calcium, was added to the production of Polish gingerbread to solve the eggshell waste problem and to reduce the risk of osteoporosis [14]. Gingerbread enriched with chicken eggshell calcium and antioxidants from green tea, added to white chocolate covering gingerbread cakes, improved the amounts of ash and calcium without significantly changing their antioxidant and sensory properties.

Special attention in gingerbread production should be paid to applying spices, such as ginger, cinnamon, cloves, and nutmeg, containing high amounts of antioxidant compounds that help neutralize harmful free radicals and reduce oxidative stress linked to aging and various chronic diseases. Our previous studies indicated that ethanolic extracts of ginger rhizome and nutmeg obtained from the ground spice powder sieved by a smaller mesh size generally had higher total levels of antioxidants determined by various analytical methods: 2,2-diphenyl-1-picrylhydrazyl (DPPH) and 2,2′-azino-bis(3-ethylbenzothiazoline-6-sulfonic acid) (ABTS), and Folin–Ciocalteu (F–C) [15]. Moreover, the ginger rhizome extracts had higher contents of phenolic acids: vanillic, ferulic, gallic, and p-OH-benzoic acids than the nutmeg extracts, in which protocatechuic, vanillic, and ferulic acids dominated. Apart from those types of spices, clove and cinnamon are also essential ingredients in gingerbread recipes. Among the studied spices widely used in gingerbread formulations, clove extracts prepared using conventional and green solvents had the highest antioxidant features [16]. In this study, three deep eutectic solvents (DESs) formed by mixing a quaternary ammonium salt-choline chloride (ChCl) with a high melting point as a hydrogen bond acceptor (HBA) and a proper mole fraction of a hydrogen bond donor (HBD), such as urea, glycerol, and propylene glycol, were applied for extraction of total antioxidants from cinnamon, nutmeg, clove, ginger, cardamom, and coriander. Furthermore, flavonoids, including rutin, hesperidin, neohesperidin, naringenin, naringin, quercetin, hesperetin, and chrysin, were comprehensive extracted from spices (mustard, rosemary, and black pepper), fruits (cranberry, fruits of *Lycium barbarum* L., grape, plum, and orange peel), and vegetables (onion and broccoli), using 17 types of DESs based on ChCl, acetylcholine chloride, choline tartrate, betaine, and carnitine with different compositions [17]. However, coumarin present in cinnamon-containing foods such as cereals, biscuits, and fine bakery products was extracted using mixtures of ChCl:L-LacA (1:2, 1:3, 1:4, 1:5), ChCl:L-LacA:1,3-propanediol (1:2:1) and solid–liquid phase extraction with conventional stirring and ultrasonic-assisted [18].

On the other hand, various hydrophilic and hydrophobic DESs as well as extraction techniques provided satisfactory results for most of the elements and heavy metals determined by plasma-based methods and atomic absorption spectrometry with low detection and quantification limits in different food products (vegetables, fruits, honey, chocolate, biscuits, dairy products (milk, dough, and cheese), tea samples, coffee products, fruit juices, wines, rice, sesame, corn, wheat, mushrooms, soya, eggs, fish, chicken meat, oils and animal fat) [19,20,21]. The advantages of element extraction utilized DESs from food matrices, compared to traditional acid digestion, were reduction in the sample amount and a shorter sample preparation time. Importantly, the use of green solvents such as DESs for element separation, instead of nitric acid and hydrogen peroxide in wet digestion, reduces accident risks and follows the principles of green analytical chemistry.

However, to the best of our knowledge, there were no references on the utilization of DESs as green extraction solvents for the extraction of elements from gingerbread.

Only some researchers [2,14,22] proposed wet and dry digestion procedures for the preparation of gingerbread samples before element determination by inductively coupled plasma-optical emission spectrometry (ICP-OES) and atomic absorption spectrometry (AAS).

For this reason, the present study focuses on the development of DESs as green solvents to extract macro- and micronutrients, heavy metals, and antioxidants from gingerbread samples. Specifically, three DESs prepared with xylitol (Xyl) and malic acid (MalA), choline chloride (ChCl) and MalA, and ChCl and lactic acid (LacA) were characterized and utilized for the separation of the target analytes with subsequent determination by spectroscopic methods. Element profiles were analyzed by inductively coupled plasma mass spectrometry (ICP-MS), while total antioxidant capacity (AC) was determined by 2,2′-azinobis(3-ethylbenzothiazoline-6-sulfonic acid) (ABTS), 2,2-diphenyl-1-picrylhydrazyl (DPPH), and cupric reducing antioxidant capacity (CUPRAC) assays. The developed DESs and ultrasound-assisted extraction (UAE) can be considered an environmentally friendly alternative to wet digestion of samples before analysis of inorganic analytes. Moreover, these studies can be primary experiments to propose the gingerbread as a candidate for reference material of mass fractions of K, Na, Mg, Ca, Mn, Zn, Fe, Cu, Cd elements.

## 2. Materials and Methods

### 2.1. Reagents

All analytical reagents required for DES preparation, elemental analyses, and antioxidant capacity determination were of analytical or HPLC grade and purchased from MERCK Sp. z o.o. (Warszawa, Poland). Calibration solutions for element analyses by using an inductively coupled plasma mass spectrometer were prepared by dilution of single-element mass concentration standards of the Central Office of Measures. Certified reference material, Polish Virginia Tobacco Leaves (INCT-PVTL-6), was used as a reference material for the ICP-MS method recovery study.

### 2.2. Gingerbread Materials

Two types of gingerbread cookies (uniced gingerbread and iced gingerbread) were kindly provided by a local confectionery company. Both gingerbread types were pulverized in a laboratory mill (FW100 model, Chemland, Stargard, Poland) to obtain powder samples (particle diameter approximately 0.5 mm).

### 2.3. Preparation of Deep Eutectic Solvents

Three two-component DESs, including Xyl:MalA, ChCl:MalA, and ChCl:LacA were prepared according to a previously reported procedure with slight modifications [23]. Briefly, Xyl or ChCl were mixed with two different compounds: MalA and LacA, with the molar ratios 1:1 in separate glass beakers. The mixtures were maintained at 80 °C and stirred at 400 rpm on a magnetic stirrer for 2 h until a homogeneous and translucent solutions were formed. To decrease viscosity and facilitate extraction, 30% distilled water was added to each DES. The synthesized DESs were stored in the dark at room temperature in closed glass bottles. The list of prepared DESs is displayed in Table 1.

### 2.4. Preparation of Gingerbread Samples

#### 2.4.1. Ultrasound-Assisted Extraction of Elements and Antioxidants Using Deep Eutectic Solvents

The extraction of elements including macro- (K, Na, Mg, Ca) and micronutrients (Mn, Zn, Fe, Cu), toxic cadmium, and antioxidants from the dried powder of ground uniced and iced gingerbread cookies with three synthesized DESs was performed using the ultrasonic bath Sono Swiss SW 6H ultrasound bath (Labo Plus, Warszawa, Poland) with a frequency of 37 KHz and ultrasound power of 540 W according to the methodology described in our previous work [23]. Based on preliminary experiments, the following solid–liquid extraction parameters were fixed: water content in each DES = 30%, liquid-to-solid ratio = 6:1 mL/g, temperature = 50 °C, and time = 15 min. Initially, the powder of two gingerbread types (5 g) was weighed into glass bottles using an analytical balance, and 10 mL of each DES was added. These mixtures were extracted for 5 min at 50 °C. The extraction was repeated three times for each sample, yielding 30 mL of each gingerbread extract. The obtained extracts were centrifuged for 5 min at 4500 rpm using a laboratory centrifuge (MPW-150R, MPW MED. INSTRUMENTS, Warszawa, Poland) and stored in a refrigerator until further analysis.

#### 2.4.2. Ultrasound-Assisted Extraction of Antioxidants Using Conventional Solvents

In this study, 70% methanol and 70% ethanol, which are conventional organic solvents, were used as comparators for the preparation of gingerbread samples before their AC determination. Separately, the UAE was performed using the same procedure as the DES-based extraction to obtain methanolic and ethanolic extracts. Briefly, an amount of 5 g of each pulverized sample together with 30 mL of each alcohol (3 × 10 mL), to give a liquid-to-solid ratio of 6:1 mL/g, was treated with ultrasonication (37 kHz and 540 W) at 50 °C for 15 min (3 × 5 min). Then, the mixtures were centrifuged at 4500 rpm for 5 min, and the obtained supernatants were stored at 4 °C until analysis.

#### 2.4.3. Microwave-Assisted Acid Digestion

Microwave-assisted acid digestion (MW-AD) was used as a reference method for the determination of inorganic elements in two types of gingerbread samples, and as a comparative method for the proposed extraction procedure employing synthesized DESs. Microwave mineralization was performed in a closed Anton Paar Multiwave Pro microwave system in an 8NXF100 rotor (Anton Paar GmbH, Graz, Austria) according to a previously reported procedure [23]. In brief, each powdered gingerbread sample (0.2 g) was digested in a closed Teflon vessel, using 6 mL of nitric acid (1:1 *v*/*v*) and 2 mL of hydrogen peroxide (30%), with the following two stages: (1) 15 min to a power of 800 W and (2) 40 min at a constant power of 800 W, max. temperature of 200 °C. After digestion, the samples were diluted to 50 mL with deionized water. A blank sample and a sample of certified reference material were prepared in parallel.

### 2.5. Analytical Methods

#### 2.5.1. Fourier Transformed Infrared (FTIR) Spectroscopy

The components of three DESs (Xyl, ChCl, MalA, and LacA) and the obtained green solvents (Xyl:MalA, ChCl:MalA, and ChCl:LacA) used in the UAE of elements and antioxidants were characterized by attenuated total reflectance—Fourier transform infrared spectroscopy (ATR-FTIR).. The ATR-FTIR spectra were obtained on a Bruker VERTEX 70v FTIR spectrometer (Bruker Optics, Ettlingen, Germany) equipped with a diamond ATR mode, with a spectral resolution of 4 cm^−1^ and an average accumulation of 64 scans. The samples were directly placed on the ATR crystal surface of the ATR probe and analyzed from 4000 to 400 cm^−1^.

#### 2.5.2. UV-Vis Spectrophotometry

The AC of uniced and iced gingerbread extracts obtained using three different DESs, 70% methanol (70% MeOH), and 70% ethanol (70% EtOH), was evaluated by previously described ABTS, DPPH, and CUPRAC spectrophotometric methods with slight modifications [24].

Briefly, in cuvettes, 0.02–0.10 mL of the prepared extracts were thoroughly mixed with respective volumes (2.48–2.40 mL) of ABTS cation radical solution (c = 7 mmol/L) to a total volume of 2.50 mL. Next, the obtained mixtures were incubated at 30 °C for 1 min, and the absorbance was measured at 734 nm.

The reduction power of DPPH radical was applied as another method for assessing the radical scavenging capacity of the gingerbread extracts. In cuvettes, the final reaction mixtures (2.50 mL) contained 0.02–0.10 mL of extracts, 1.98–1.90 mL of methanol, and 0.5 mL of working DPPH radical solution (c = 304.0 μmol/L). After 15 min of reaction at ambient temperature in the dark, absorbance was read at 517 nm.

In the case of the CUPRAC assay, 0.1–3.0 mL of the studied extracts were taken to 10 mL calibration flasks and 2 mL of copper chloride solution (c = 0.01 mol/L), neocuproine solution (c = 7.5 mmol/L), ammonium acetate buffer (pH = 7.26), and made up to volume with deionized water. At the end of 30 min of reaction time in a dark place, absorbance was measured at 450 nm.

Calibration curves were prepared using working solutions of Trolox (TE) in methanol between 3.00 × 10^−5^ to 1.50 × 10^−4^ mmol TE/mL, 2.00 × 10^−5^–1.00 × 10^−4^ mmol/mL, and 1.00 × 10^−5^–6.00 × 10^−5^ mmol TE/mL for ABTS, DPPH, and CUPRAC methods, respectively. The least-squares method was utilized to calculate the regression lines: %ABTS = (354,949 ± 13,510) × c_TE_ (mmol/mL) + (8.71 ± 1.26), %DPPH = (609,575 ± 15,664) × c_TE_ (mmol/mL) + (3.39 ± 1.02), and Abs_450_ = (13,459 ± 195) × c_TE_ (mmol/mL) − (0.0073 ± 0.0076) resulting in determination coefficients (R^2^), 0.9914, 0.9954, and 0.9992, respectively.

The absorbance of the prepared solutions in five repetitions was measured with a Hitachi U-2900 spectrophotometer (Tokyo, Japan).

The AC of the studied gingerbread samples was given as mmol Trolox equivalent (TE) per 100 g in all assays.

#### 2.5.3. Inductively Coupled Plasma Mass Spectrometry

The ICP-MS methodology reported previously [23] was applied to quantify inorganic analytes (K, Na, Mg, Ca, Mn, Zn, Fe, Cu, and Cd). Briefly, gingerbread samples after UAE-DESs and MW-AD were introduced into an inductively coupled plasma mass spectrometer (ICP-MS Agilent 7800, Agilent Technologies, Inc., Tokyo, Japan) equipped with a nebulizer (gas flow = 1.01 L/min) and spray chamber (temperature = 2 °C), operating at RF power (1550 W) in plasma generation (15.0 L/min), and auxiliary gas (0.90 L/min). The isotopes (internal standard isotopes) ^23^Na (^73^Ge), ^39^K (^45^Sc), ^44^Ca (^45^Sc), ^24^Mg (^89^Y), ^55^Mn (^115^In), ^66^Zn (^115^In), ^56^Fe (^73^Ge), ^63^Cu (^115^In), and ^111^Cd (^115^In) were selected as analytical masses in ICP-MS through which helium was flowing as collision gas (5 mL/min). Samples after extraction required 20-fold dilution with 1% nitric acid due to high viscosity.

### 2.6. Statistical Analysis

AC determination of all gingerbread extracts was carried out in five-fold, while the element profile for each prepared sample after extraction using three DESs and wet digestion was repeated twice. All results are expressed as mean value ± standard deviation (SD). Significant differences between means were determined by one-way analysis of variance (ANOVA) followed by Duncan’s post hoc test (*p* < 0.05). The relationships between determined elements and AC results were assessed using Pearson’s correlation analysis and mapped using a color matrix. Hierarchical cluster analysis (HCA) was also performed to assess the similarities and discrepancies between macro- and micronutrients, heavy metal concentrations and antioxidant potential of uniced and iced gingerbread extracts prepared in green solvents. Data analysis was conducted using the IBM SPSS ver. 9 PS IMAGO PRO Academic (institutional license purchased by the Nicolaus Copernicus University in Toruń, Toruń, Poland) and the Statistica 8.0 software (StatSoft, Tulsa, OK, USA). Additionally, to assess and contrast the greenness of gingerbread sample preparation techniques before determination of element profiles and antioxidant potential the AGREEprep software (v. 0.91) (Analytical GREEness for Sample Preparation, available for free [25] was employed.

## 3. Results and Discussion

### 3.1. Characterization of the Synthesized Deep Eutectic Solvents

**Figure 2:**
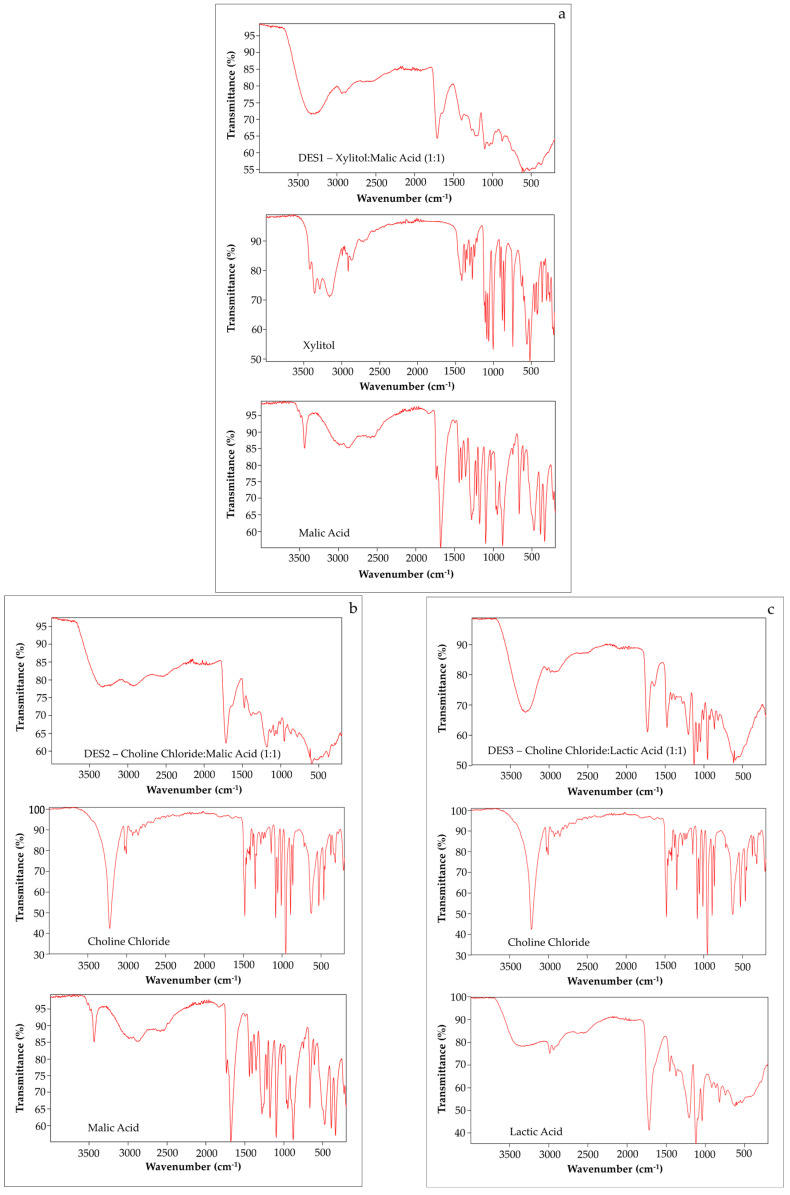
ATR-FTIR spectra of synthesized DESs, (**a**) xylitol–malic acid, (**b**) choline chloride–malic acid, (**c**) choline chloride–lactic acid, and their initial reagents.

The obtained DESs and their constituting components (HBA and HBD) listed in Table 1 were characterized by ATR-FTIR spectroscopy. The synthesis of each DES was confirmed by the occurrence of hydrogen bonding between the carboxylic and hydroxyl groups of the initial compounds, together with intermolecular interaction (Van der Waals forces, and electrostatic interactions) [26,27]. The ATR-FTIR spectra (Figure 2) demonstrated the presence of these functional groups in precursors of the studied DESs, and characteristic interactions confirmed the DES conversion.

Some similar bands on the recorded spectra of the synthesized DESs were found, and their shift was due to the characteristic self-assembly of the HBAs and HBDs, resulting in eutectic mixtures. These interactions could be observed by the shifts and broadening of the intense bands in the range between 3700 and 3100 cm^−1^, corresponding to the −OH stretching vibration. Additionally, the characteristic peak at about 1630 cm^−1^ can be attributed to angular deformation of the −OH groups of the water molecules added to all prepared DESs to decrease their viscosity. Furthermore, the C=O stretching band of each DES appeared at higher wavelengths (above 1700 cm^−1^) in comparison with the spectra of DES precursors (Figure 2). It indicates the formation of hydrogen bonds in these green solvents as evidenced by increased electron density of the carbonyl oxygen. Other authors recorded similar FTIR spectra of the proposed DESs with characteristic bands, confirming their successful synthesis [26,27,28].

The spectrum of malic acid used as initial compound for preparation of DES1 and DES2 presented a peak at 3437 cm^−1^ ascribed to −OH stretching vibration, together with a C=O stretching broad band with two maxima at 1680 and 1738 cm^−1^, as well as a band at 1178 cm^−1^ associated with the CH_2_ stretching band [27,28]. However, broadband observed in the 3450–3150 cm^−1^ in the xylitol spectrum, a five-carbon sugar alcohol utilized in DES1 preparation, corresponds to free −OH groups in the molecule, whereas bands at 1431 and 743 cm^−1^ were assigned to the in-plane and out-of-plane flexions of −OH, respectively. Additionally, a broad absorption band between 3000 and 2800 cm^−1^ ascribed to C-H stretching vibration, C=O stretching vibration at 1638 and 1713 cm^−1^, and C-O stretching vibration at 1005, 1063, 1087, and 1111 cm^−1^, were recorded on the spectrum of xylitol (Figure 2a) [29,30]. Nevertheless, changes including the shifted peak from 1680 to 1713 cm^−1^ ascribed to C=O and a broad peak in 3100–3600 cm^−1^ assigned to the −OH group can be observed in the ATR-FTIR spectra of two precursors and the obtained DES1 (Figure 2a).

On the other hand, the ChCl spectrum presented characteristic absorption bands at 3219 cm^−1^ and 1200–880 cm^−1^ corresponding to hydroxyl or amino groups (N-H stretching and C-N+ symmetric stretching), an absorption band at 3006–2853 cm^−1^ assigned to C-H stretching vibration, and the peak at 1481 cm^−1^ referring to the C-H bending vibration (Figure 2b,c) [28,31]. Interestingly, the CH_2_ bending of an alkyl group with characteristic absorption at 1481 cm^−1^ is the prominent group detected in all ChCl-based DESs. The spectrum of DES2 containing ChCl:MalA allowed for the confirmation of the eutectic solvent formation due to the shift in the C=O stretching band from 1680 cm^−1^ and 1738 cm^−1^ to 1715 cm^−1^ (Figure 2b). Probably it is the result of the destruction of a significant part of MalA dimers and the generation of a hydrogen bond between MalA and ChCl. Moreover, spectrum changes in the band of the −OH group of MalA and the intense vibrational −OH band in ChCl at 3219 cm^−1^ to a broad band 3600–3200 cm^−1^ confirmed a new hydrogen interaction.

Figure 2c also shows spectra of ChCl-based DES3 and its precursors, including ChCl and LacA. LacA spectrum is characterized by the broad peak in the 3600–3100 cm^−1^ and 2986 cm^−1^ corresponding to −OH and −CH stretching vibrations, respectively. Moreover, a strong band at 1719 cm^−1^ is assigned to C=O stretching vibrations, while absorption bands observed at 1453 cm^−1^ and 1375 cm^−1^ are ascribed to −CH bending vibrations (Figure 2c) [28]. After the DES3 formation, a shift in the −OH stretching vibrations of LacA (3600–3100 cm^−1^) towards smaller wavenumbers (a broad peak about 3450 cm^−1^) was recorded. Moreover, the vibrational signal at 2986 cm^−1^ in the LacA spectrum corresponding to the −CH vibration shifted to 3034 cm^−1^ in a DES3 spectrum, while the absorption band corresponding to the C=O stretching was shifted from 1719 cm^−1^ (LacA spectrum) to 1729 cm^−1^ (DES3 spectrum).

It is noteworthy that the vibrational band at 1349 cm^−1^ disappeared after the synthesis of ChCl-based DESs [32,33]. The lack of new absorption bands in the region of 1650–1550 cm^−1^ and 1400 cm^−1^ of DES2 and DES3 spectra, assigned to the stretching vibrations of carboxylate groups, indicated hydrogen bonding between initial reagents and also confirmed the DES formation between ChCl and carboxylic acids [34].

### 3.2. Elemental Compositions of Uniced and Iced Gingerbread Cookies

The three synthesized DESs were applied for the UAE of macroelements (potassium, sodium, magnesium, calcium), microelements (manganese, zinc, iron, copper), and a representative toxic element (cadmium) from the uniced and iced gingerbread samples prior to their determination by using ICP-MS. The element concentrations in the obtained green extracts of two kinds of gingerbread samples were compared with those prepared using microwave-assisted acid digestion (MW-AD) and are listed in Table 2.

As shown in Table 2, the element concentrations determined in DES-based extracts (except for two micronutrients, Zn and Cu contents in DES3-based extract) were significantly lower than those in gingerbread samples after MW-AD. Less effective extraction of four macronutrients (K, Na, Mg, and Ca) and two micronutrients (Mn and Fe) by all investigated DESs can be due to the more stable complexes in the analyzed samples themselves compared with complexes formed with carboxylic acids in the DES phase. This fact confirms the complexity of the investigated matrices, in which these four macro- and two micronutrients are probably strongly bound to other organic gingerbread cake components, hindering their release into solution.

Moreover, uniced gingerbread samples prepared using acid digestion and microwave irradiation were richer sources of the studied elements (except Na) than iced gingerbread treated with wet digestion. Unexpectedly, wet-digested uniced gingerbread revealed 3 times lower sodium concentration (590.5 mg/kg) in comparison with iced gingerbread mineralizate (1859.9 mg/kg). This may be due to the fact that confectionery manufacturers add sodium chloride to confectionery products, mainly to sugar-based icing. It is well-known that the saltiness of higher sodium chloride concentrations is known to mask the sweetness of sucrose, whereas low sodium chloride concentrations can also have a slight sweet taste [35].

On the contrary, DES-based extracts of iced gingerbread contained significantly higher amounts of macro- (K, Na, Mg, Ca) and micronutrients (Mn, Zn, Fe, Cu) than the same extracts of uniced gingerbread (Table 2, Duncan test). This suggests that higher sucrose content in sugar-based icing after contact with three DESs creates new green solutions with more effective recoveries of inorganic analytes. A stronger hydrogen bond was probably formed due to the increase in the amount of sucrose. For this reason, the natural DES containing citric acid (CitA) and a higher content of sucrose (1:3) was selected for the successful microextraction of lead and cadmium from different vegetables [36].

The results of element profiles for two types of gingerbread cookies confirm that the extraction properties of the proposed DESs depend on the nature of the HBD and HBA. As can be seen, two ChCl-based green solvents (DES2 and DES3) showed a higher efficiency in the extraction of micronutrients (Mn = 0.26–2.07 mg/kg, Zn = 2.41–28.66 mg/kg, Fe = 2.96–9.86 mg/kg, and Cu = 0.56–3.45 mg/kg) compared to micronutrients amounts (Mn = 0.29–1.47 mg/kg, Zn = 1.40–1.92 mg/kg, Fe = 1.07–2.52 mg/kg, and Cu = 0.42–1.48 mg/kg) in extract obtained by using DES1. An insignificant difference in Cu concentration was also observed between the extract of uniced gingerbread prepared using DES2 and mineralizate after MW-AD (Table 2, Duncan test). Moreover, Cu content in the DES1-based extract and the sample after wet digestion was similar. Unexpectedly, DES3-based extracts of two types of gingerbread had significantly higher Cu (1.05–3.45 mg/kg) and Zn (12.19–28.66 mg/kg) concentrations than those in samples after MW-AD (Cu = 0.48–0.58 mg/kg and Zn = 5.01–5.63 mg/kg). Probably, LacA as a component of DES3 strongly chelated and solubilized Zn and Cu ions from cake matrices due to the formation of hydrogen bonding or ionic interactions, causing more efficient extraction of these two analytes compared to acid digestion. On the other hand, wet digestion requires the use of aggressive oxidants and concentrated reagents, which can lead to carbonisation, volatilisation, and loss of some inorganic analytes, negatively affecting the overall experimental procedure.

Similarly, the concentrations of Cu (9.54–36.97 μg/g and 10.19–37.55 μg/g) and Zn (31.22–936.00 μg/g and 31.52–940.12 μg/g) determined by AAS and ICP-OEC, respectively, in ChCl-oxalic acid-based extract from the different parts (muscle, liver, and gills) of a marine fish sample were higher than the amounts of Cu (0.00–27.49 μg/g) and Zn (29.42–889.63 μg/g) obtained after conventional acid digestion and determination by AAS method [37].

Among the carboxylic acids, LacA, as a HBD component of ChCl-based DES3, provided more effective extraction of micronutrients than MalA used to generate DES2 (Table 2). In opposite, the use of DES1 and DES2 containing MalA resulted in satisfactory recoveries for macronutrients (K = 95.0–532.0 mg/kg, Na = 51.1–627.8 mg/kg, Mg = 40.1–101.0 mg/kg, Ca = 10.8–81.3 mg/kg). The presence of MalA in DES1 and DES2 greatly affected the extraction of singly charged elements such as sodium and potassium from gingerbread samples. This can be explained by the fact that the hydroxide groups of carboxylic acids can generate chelate complexes with cations, which is a main driving force for effective mass transfer. The extraction potential of these green solvents is probably associated with the capacity of the prepared DESs to form hydrogen bonds with the investigated inorganic analytes. DES acts as a Lewis acid when in the presence of the electron-donating group of the sample, extracting the inorganic element from the sample to the DES solution [38].

Importantly, cadmium, a toxic, mutagenic, and carcinogenic element, was not detected in gingerbread cookies prepared using UAE-DESs and MW-AD.

Ivanišová et al. [39] determined similar levels of microelements, including Mn (5.02 mg/kg), Zn (6.39 mg/kg), Fe (13.82 mg/kg), and Cu (1.90 mg/kg) in control gingerbread containing conventional beet sucrose as an ingredient, using microwave digestion and AAS. However, the replacement of refined beet sucrose in gingerbread formulation with different types of sweeteners (cane sugar, sorbitol, xylitol, maple syrup) significantly affected the amounts of these micronutrients (Mn = 1.69–2.10 mg/kg, Zn = 6.19–7.41 mg/kg, Fe = 16.00–21.40 mg/kg, and Cu = 1.69–2.10 mg/kg). Moreover, both partial and total replacement of honey with sugar beet molasses (10–40%) resulted in a prominent increase in macro- and microelements (K = 1504 mg/kg and 4091–11,773 mg/kg, Mg = 100 mg/kg and 200–400 mg/kg, Ca = 500 mg/kg and 700–1120 mg/kg, Fe = 19.4 mg/kg and 24.3–45.2 mg/kg before and after replacement, respectively) in gingerbread biscuits due to the high concentration of these minerals in molasses [2]. Nevertheless, manganese and zinc contents did not vary over the analyzed biscuits, whereas copper was lower in the biscuits with molasses. Similarly, the changes in the original gingerbread cake recipe by introducing chickpea flour, bean flour, and sugar beet powder increased concentrations of potassium (from 1222.4 mg/kg to 2023.5–3465.2 mg/kg), calcium (from 201.7 mg/kg to 185.9–432.7 mg/kg) and iron (from 10.3 mg/kg to 17.2–23.6 mg/kg), creating a new range of gingerbread products [40]. Interestingly, Polish gingerbread was enriched with chicken eggshell and green tea powder to increase the calcium and antioxidant intake, and solve the eggshell waste problem [14]. The calcium levels were 867.1 and 7294.2 mg/kg for gingerbread without and with 3% eggshell powder after dry mineralization in a muffle furnace and AAS determination.

However, there have not been studies concerning utilizing DESs to extract inorganic elements from gingerbread cookies. Although DESs based on Xyl, MalA and CitA were used as promising solvents in UAE of tomato leaf, spinach leaf and forage grass samples before the determination of K, Na, Mg, Ca, Mn, Zn, Fe, Cu, and Cd by ICP-OES and ICP-MS [26]. The acceptable ranges of recoveries (80–114%) were obtained for Cd, K, Mn, and Zn (in Mal:Xyl extract of tomato leaf), Cd, K, Mn, Na, and Zn (in Mal:Xyl extract of spinach leaf), and Ca, Cd, Fe, K, Mn and Mg (in Mal:Xyl extract of forage samples). Additionally, a DES system based on ChCl and LacA (1:1) had a higher extraction efficiency of Cd (90.8%), Cu (80.0%), Zn (83.6%), and Fe (86.3%) from litterfall than ChCl:MalA (1:1) (90.0, 82.0, 79.6, and 83.4% for Cd, Cu, Zn, and Fe, respectively) [41].

The element profile results showed that the prepared DESs containing carboxylic acids are promising green solvents for the simultaneous extraction and separation of singly- and multiply charged elements from gingerbread samples. However, further studies are needed to optimize the HBD nature, DES component ratio, DES and sample ratio, and other extraction conditions, including temperature and time, for the more effective extraction of inorganic analytes from the food matrices. The choice of green solvents is a preliminary experiment in the development of gingerbread cake as a candidate for reference materials with a dedicated content of macro-, microelements and heavy metals.

### 3.3. Antioxidant Capacity of Uniced and Iced Gingerbread Cookies

In this study, the AC of uniced and iced gingerbread cookies extracts prepared using three DESs and conventional well-known solvents, 70% MeOH and 70% EtOH, were evaluated by three different modified spectrophotometric assays: ABTS, DPPH, and CUPRAC and the obtained results are presented in Table 3.

As can be observed, there were significant differences in the AC values between DES-based extracts and those obtained with two alcohols (Duncan test, Table 3). Surprisingly, conventional solvents extracted antioxidants more efficiently than the proposed green solvents, which resulted in higher AC results determined by all proposed analytical methods. All DES-based extracts had a lower ability to scavenge ABTS and DPPH radicals (lower antiradical capacity) than methanolic and ethanolic extracts. Although insignificant differences in CUPRAC values were found between DES3-based extracts and methanolic extracts of gingerbread samples (Duncan test, Table 3). This suggests that the green solvent created by combining ChCl and LacA at the molar ratio (1:1), as well as 70% MeOH, provided a comparable extraction recovery of antioxidants with the highest ability to redox reaction and form Cu(I)-neocuproine chelate. Additionally, the Duncan test indicated that the AC of methanolic and ethanolic gingerbread extracts measured using all analytical assays did not differ significantly. However, ethanolic extracts revealed somewhat higher AC results than methanolic extracts. This can be explained by the fact that gingerbread samples contained higher amounts of relatively hydrophobic antioxidants because ethanol with a lower dielectric constant (25.16) is a more effective conventional solvent to extract less polar compounds in comparison with methanol, characterized by a higher dielectric constant (33.64) [42].

Moreover, the AC results demonstrated significant differences between ABTS and CUPRAC values for DES1 (0.59–1.22 and 0.04–0.71 mmol TE/100 g), DES2 (1.05–3.28 and 0.06–1.17 mmol TE/100 g), and DES3-based (0.84–1.20 and 1.12–1.54 mmol TE/100 g) extracts of uniced and iced gingerbread cookies (Duncan test, Table 3). Evidently, the presence of MalA in DES1 and DES2 solvents enhanced the recovery of antioxidants from iced gingerbread samples capable of scavenging ABTS cation radicals and reducing the CUPRAC reagent. In contrast, these two DESs proved ineffective in the extraction of bioactive molecules quenching DPPH radicals. However, DES3, composed of ChCl and LacA, was the most suitable green solvent for efficient extraction of the hydrophilic and hydrophobic antioxidants from gingerbread samples, which simultaneously could deactivate ABTS and DPPH radicals and be associated with Cu(II)−Cu(I) reduction (Table 3). Generally, DES3 extracted significantly more antioxidants from uniced gingerbread cookies (ABTS = 1.20 mmol TE/100 g, DPPH = 0.71 mmol TE/100 g, and CUPRAC = 1.54 mmol TE/100 g) than from iced gingerbread samples ABTS = 0.84 mmol TE/100 g, DPPH = 0.75 mmol TE/100 g, and CUPRAC = 1.12 mmol TE/100 g). This suggests that additional amounts of sugar in icing affected DES3 efficiency and effectiveness in extracting antioxidant compounds from the studied samples.

Despite the lower AC for DES-based extracts of two types of gingerbread, these eco-friendly solvents represent a new, valuable alternative to alcohols used as conventional solvents. Generally, the possibility of DES design permits the composition of green solvents with lower vapor pressure and higher solvating capacity, less toxic, more selective, and lower energy consumption; therefore, they can recover thermolabile and sensitive compounds from the complex matrices [43].

Among the proposed DESs, those containing ChCl as HBA have been more effective for the extraction of both small bioactive molecules and biomacromolecules. However, two carboxylic acids (MalA and LacA) as HBD components have been widely used for extraction of bioactive compounds due to their hydrophilic character with pKa of 3.5 to 4.5 [43,44]. Therefore, the synthesis of new DESs as prospective and valuable solvents requires knowledge about the structure of extracted antioxidant compounds due to the principle of dissolving “like dissolves like”. Furthermore, the −OH groups present in the chemical structure of antioxidants can form intermolecular hydrogen bonds with DESs, creating stable and robust dynamic networks and increasing their extraction from the various matrices [44].

Recently, many researchers have applied various DESs, including betaine:D,L-LacA:H_2_O; MalA:glucose (30% H_2_O), betaine:ethylene glycol (30% H_2_O), betaine:sucrose (30% H_2_O) and different extraction parameters (time, temperature, sonification amplitude/ultrasound power) to extract antioxidant components from ginger as a main spice added to gingerbread formulations [45,46,47]. Similarly, like in this work, the ABTS values (5.88–6.89 µM TE/g and 15.37–19.12 µM TE/g for bound and free phenolic fraction, respectively) for gingerbread cookies without and with elderflower dry extract and elderberry juice concentrate and covered with dark chocolate were significantly higher than those obtained by the DPPH method (1.03–1.51 µM TE/g and 0.19–1.19 µM TE/g for bound and free phenolic fraction, respectively) [12]. Furthermore, the AC results of 80% ethanolic extracts of gingerbread samples baked with wheat flour and quinoa flour, and defatted apple seed cake flour in different proportions and analyzed by DPPH, FRAP and CUPRAC methods differed significantly [6]. The obtained CUPRAC values (2.08–3.90 mg equivalents of ascorbic acid per g of dry weight sample (mg AAE/g DW)) were significantly higher than FRAP (0.72–0.92 mg AAE/g DW) and DPPH (1.05–1.19 mg TE/g DW) results.

### 3.4. Greenness Assessment of the Used Analytical Procedures

**Figure 3:**
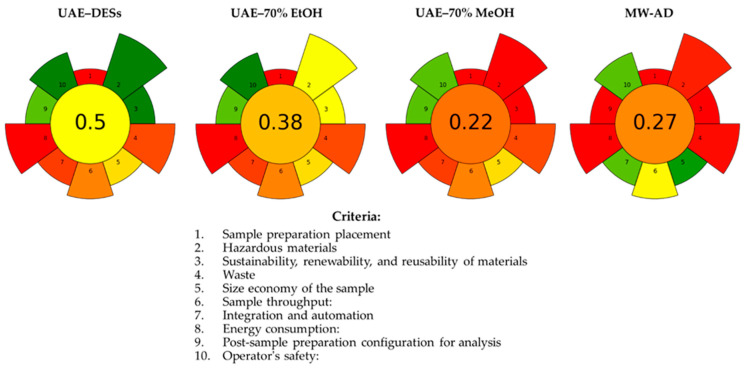
Comparison of the AGREEprep metrics for ultrasound-assisted extraction of elements and total antioxidants from gingerbread cookies using three DESs (UAE-DESs), 70% ethanol (UAE-EtOH), 70% methanol (UAE-MeOH) and microwave-assisted acid digestion (MW-AD). The total score ranked from 0 to 1 (the central, circular field corresponds to the final assessment score), and the 10 criteria were studied with a scaling of the criterion’s weight (1 to 5) against the total value in the form of segment size and value obtained on a scale from red to green.

The AGREEprep tool was applied to assess the greenness of gingerbread sample preparation techniques before the determination of element profiles and antioxidant potential based on ten key criteria, including reagent safety, waste reduction, energy efficiency, and operator safety. The generated AGREEprep metrics were depicted as colored pictograms (Figure 3).

This metric tool is based on a scoring system ranging from 0 to 1, along with a red-yellow-green scale indicating weaknesses and strengths of the applied analytical procedures. Considering the ecological aspect of environmental sustainability, the proposed DES-based extraction provided the best scores (0.50) according to the AGREEprep metric (Figure 3). The extraction of inorganic analytes and antioxidants by new synthesized DESs involved replacing hazardous reagents with eco-friendly solvents. However, the weaker points of the DES-based extraction were the lack of automation, which could affect sample throughput, operational efficiency, and high energy consumption. Despite these disadvantages, the overall environmental impact of UAE-DES procedures is much lower than that of traditional sample preparation.

In contrast, the UAE-70% MeOH and MW-AD techniques had the lowest points (0.22 and 0.27, respectively), attributed to the use of toxic methanol and nitric acid, the generation of harmful waste, and concerns over operator safety. Consequently, the change of 70% methanol to 70% ethanol as a less toxic and more environmentally friendly solvent for ultrasonic extraction of antioxidants from gingerbread samples significantly increased the total score (0.38) of this procedure (Figure 3).

On the other hand, MW-AD had a higher analytical frequency, because 8 samples can be digested simultaneously, while the UAE can only handle three samples in 1 h.

The investigated UAE-DES methodology strongly aligns with the principles of green analytical chemistry, making it a sustainable and practical choice for routine analysis of elements and antioxidants in gingerbread cookies.

The similar scores of 0.54, 0.45, and 0.28 were obtained for the microwave-assisted extraction (MAE) and UAE employing DESs (CitA:Xyl and CitA:MalA), and MW-AD using diluted nitric acid applied before determining As, Cd, and Pb in plant and food matrices by the ICP-MS method [48]. However, a higher overall score of 0.74 was calculated for UAE of phenolic compounds from aloe vera rind by-product using ChCl:CitA as a green solvent before HPLC analysis with a photodiode array detector [49].

### 3.5. Chemometric Analysis

#### 3.5.1. Correlation Analysis Between Elemental Analysis and Antioxidant Capacity

**Figure 4:**
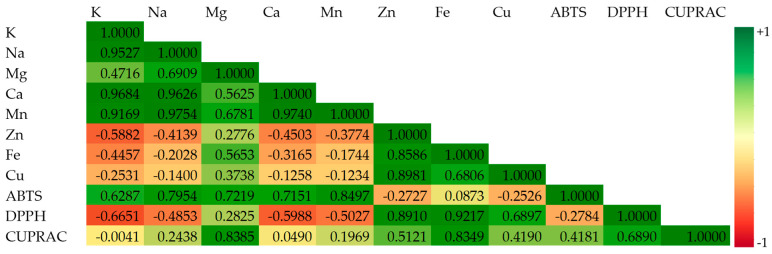
Color matrix showing Pearson correlation analysis between concentrations of macro- and microelements and antioxidant capacity of DES-based extracts of uniced and iced gingerbread cookies.

The relationships between the concentrations of macro- and micronutrients in DES-based extracts of two types of gingerbread cookies and their antioxidant properties, determined by three analytical methods (ABTS, DPPH, and CUPRAC), are depicted in Figure 4 as a color matrix.

Importantly, this color matrix illustrated positive associations between CUPRAC-DPPH (r = 0.6890, *p* = 0.1301) and CUPRAC-ABTS (r = 0.4181, *p* = 0.4094) results, while ABTS did not correlate with DPPH values (r = −0.2784, *p* = 0.5933). Probably, prepared DESs offered low efficiency in the extraction of hydrophobic antioxidants that interacted with the DPPH and ABTS free radicals, causing their extinction.

It can be noted that DPPH (r = 0.6897–0.9217, *p* = 0.0090–0.1295) and CUPRAC (r = 0.4190–0.8349, *p* = 0.0386–0.4083) results for the studied green extracts were positively correlated with three micronutrients (Zn, Fe, and Cu), while ABTS values were significantly and positively associated with all analyzed macronutrients (r = 0.6287–0.7954, *p* = 0.0585–0.1812) and manganese (r = 0.8497, *p* = 0.0322). This can be explained by the fact that at the same time, the synthesized DESs can extract Zn, Fe, Cu ions and antioxidants capable of scavenging purple DPPH free radicals and create the orange Cu(I)-neocuproine complex. However, the presence of macroelements and manganese in the investigated extracts enhanced radical scavenging activity against ABTS cation radicals. High and significant correlation coefficient (r = 0.8385, *p* = 0.0370) between magnesium level and CUPRAC indicates the effectiveness of the proposed DESs for simultaneous extraction of magnesium and hydrophilic and hydrophobic antioxidants with reducing ability of copper(II)-neocuproine chelate. The positive correlations between antioxidant properties and inorganic analytes indicate that concentrations of some antioxidants increased with increasing contents of certain minerals. This phenomenon can be explained by the fact that antioxidants interact synergistically with minerals.

On the contrary, higher amounts of K, Na, Ca and Mn in prepared extracts demonstrated inverse correlations with DPPH values (r = −0.4853–−0.6651, *p* = 0.1494–0.3292), suggesting their important role in the possible inhibition of antioxidants to scavenge DPPH radicals.

Additionally, there were high positive associations between all studied macronutrients (K, Na, Mg, and Ca) and manganese (r = 0.4716–0.9754, *p* = 0.0009–0.3450). It was speculated that the synthesized DESs had similar efficiency to extract K, Na, Mg, Ca, and Mn. Moreover, zinc exhibited significant positive relationships with iron and copper (r = 0.8586, *p* = 0.0286 and 0.8981, *p* = 0.0150 for Zn–Fe and Zn–Cu, respectively). Only a somewhat lower correlation coefficient (r = 0.6806, *p* = 0.1367) was calculated between iron and copper in the evaluated DES-based gingerbread extracts. Unfortunately, low and negative correlation coefficients between the analyzed macroelements and microelements confirm the necessity of selecting different green solvents to extract macronutrients and micronutrients from gingerbread samples.

#### 3.5.2. Hierarchical Cluster Analysis

**Figure 5:**
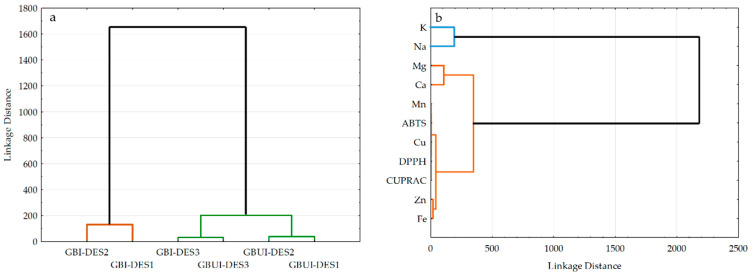
Dendrograms of hierarchical cluster analysis for (**a**) six DES-based extracts of uniced (GBUI) and iced (GBI) gingerbread samples and (**b**) the studied variables: concentrations of macro- (K, Na, Mg, Ca) and micronutrients (Mn, Zn, Fe, Cu), and antioxidant features determined by ABTS, DPPH, and CUPRAC assays.

HCA was employed to evaluate the similarities among six DES-based extracts of gingerbread cookies based on variables, such as concentrations of macro- and micronutrients determined by the ICP-MS method and antioxidant potential analyzed using modified ABTS, DPPH, and CUPRAC assays. This cluster analysis rendered dendrograms depicted in Figure 5 grouping the DES-based extracts of uniced (GBUI) and iced (GBI) gingerbread samples (Figure 5a) and all studied analytes (K, Na, Mg, Ca, Mn, Zn, Fe, Cu, ABTS, DPPH, and CUPRAC) (Figure 5b).

Notably, the six extracts of gingerbreads were classified into two main clusters according to their element profiles and antioxidant features (Figure 5a, Table 2 and Table 3). The first group was clearly distinguished because two extracts of iced gingerbread prepared using DES1 and DES2 were the richest in macronutrients (K, Na, Mg, and Ca), manganese, and total antioxidants measured by ABTS assay. The second group, including all green extracts of uniced gingerbread and iced gingerbread prepared using DES3 was quite separated, because had a similar sodium content (51.0–69.7 mg/kg) and the lowest activity to scavenge ABTS (0.59–1.20 mmol TE/100 g) and DPPH (0.0–0.75 mmol TE/100 g) radicals. However, two extracts (GBI-DES3 and GBUI-DES3) based on ChCl:LacA arranging in this group characterized by the highest concentrations of three micronutrients (Zn = 12.19–28.66 mg/kg, Fe = 8.84–9.86 mg/kg, and Cu = 1.05–3.45 mg/kg) and the most substantial antioxidant potential measured by all analytical tests (ABTS = 0.84–1.20 mmol TE/100 g, DPPH = 0.71–0.75 mmol TE/100 g, and CUPRAC = 1.12–1.54 mmol TE/100 g). Evidently, DESs containing MalA (DES1 and DES2) can extract similar levels of macroelements (K = 95.0–128.3 mg/kg, Na = 51.1–57.7 mg/kg, Mg = 40.1–46.3 mg/kg, and Ca = 10.8–28.7 mg/kg) and the lowest contents of total antioxidants (ABTS = 0.59–1.05 mmol TE/100 g, DPPH < LOD, CUPRAC = 0.04–0.06 mmol TE/100 g) from a uniced gingerbread sample.

On the other hand, the HCA indicated that the determined elements and AC comprised two main groups (Figure 5b). As can be seen, two macroelements (K and Na) with the highest levels in the studied gingerbread samples formed the first cluster. However, the second group was composed of two subgroups: I—Mg and Ca and II—all micronutrients (Mn, Cu, Zn, and Fe) and AC determined by all analytical assays (ABTS, DPPH and CUPRAC). Generally, Zn and Fe formed the second subgroup in this cluster, connected with Cu. This can be explained by the significant impact of these three microelements on simultaneously quenching DPPH radicals and reducing Cu(II)-neocuproine complex by the antioxidants present in the investigated extracts (high values of correlation coefficients, r = 0.6897–0.9217 and 0.4190–0.8349 for relationships between Zn, Fe, Cu—DPPH and Zn, Fe, Cu—CUPRAC, respectively, Figure 4). In addition, the distinct subcluster containing Mn and ABTS confirms that the manganese amount in gingerbread cookies significantly affected ABTS results, enhancing decolorization of ABTS cation radicals by the extracted antioxidants (r = 0.8497, Figure 4).

The HCA results demonstrated that formulations of gingerbread cookies and applied green solvents for the preparation of extracts had a significant impact on their element profiles and antioxidant features. These findings support the potential development of healthy gingerbread cakes and certified materials, which provide valuable insights for controlling macro-, micronutrients and toxic metals in confectionery products in each stage of the technological process.

## 4. Conclusions

The UAE using three green DESs as an environmentally friendly, rapid and relatively inexpensive technique was applied to prepare two types of gingerbread cookies before the determination of macro- (K, Na, Mg, and Ca) and micronutrients (Mn, Zn, Fe, and Cu), cadmium, and antioxidant properties. The studied DES-based extracts of iced gingerbread cookies were a richer source of inorganic nutrients and antioxidant compounds than the uniced ones. Two MalA-based DESs (DES1 and DES2) achieved the highest extraction efficiency of four macroelements and manganese, as well as in terms of antioxidants able to scavenge ABTS cation radicals. However, microelements such as Zn, Fe, and Cu were more effectively extracted into DES3, containing ChCl and LacA. Moreover, this DES3 can be selected as the most promising solvent for the extraction of antioxidants with the capacity to reduce Cu(II)-neocuproine reagent to the highly colored Cu(I)-neocuproine chelate. Generally, DES3-based and methanolic extracts of the studied gingerbread samples have similar CUPRAC results. Importantly, toxic cadmium was not detected in the extracts of gingerbread samples prepared based on the complex formation of Cd(II) with DES components, nor in mineralized samples after microwave-assisted acid digestion. Nevertheless, the greater effectiveness of the DES3 in the gingerbread treatment for the determination of two microelements (Zn and Cu) than after acid digestion indicates different chemical interactions between these two inorganic analytes and the synthesized DESs. Therefore, the developed DES3-UAE and ICP-MS analytical methodology can be a perspective for studies on producing gingerbread reference materials with certified amounts of zinc, copper, and cadmium.

Furthermore, the proposed sample preparation using DESs also follows green chemistry recommendations regarding reducing concentrated and toxic reagents, consequently creating less aggressive waste. However, future studies are needed that focus on the optimization of extraction conditions and DES compositions with good thermal stability, low toxicity, good biodegradability, and reusability to enhance the simultaneous extraction efficiency of inorganic elements and bioactive compounds from confectionery matrices.

## Figures and Tables

**Table 1:** Composition of the prepared DESs and structural formulas of their initial components.

Abbreviation	Hydrogen Bond Acceptor (HBA)		Hydrogen Bond Donor (HBD)		Molar Ratio (mol/mol)
	Chemical Name	Structural Formula	Chemical Name	Structural Formula	
DES1-Xyl:MalA	Malic Acid	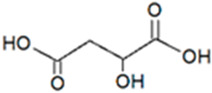	Xylitol	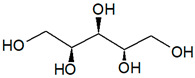	1:1
DES2-ChCl:MalA	Choline Chloride	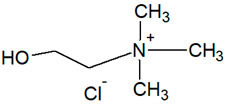	Malic Acid	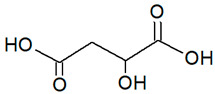	1:1
DES3-ChCl:LacA	Choline Chloride	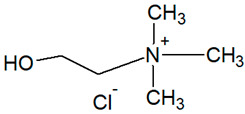	Lactic Acid	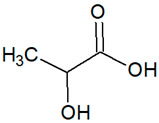	1:1

**Table 2:** Concentrations of elements in uniced and iced gingerbread cookies prepared by ultrasound-assisted extraction using three deep eutectic solvents and microwave-assisted acid digestion.

Element	Concentration * ± SD (mg/kg)							
	DES1-Xyl:MalA		DES2-ChCl:MalA		DES3-ChCl:LacA		MW-AD	
	Uniced	Iced	Uniced	Iced	Uniced	Iced	Uniced	Iced
K	95.0 ± 14.0 ^a^	532.0 ± 112.0 ^b^	128.3 ± 20.0 ^a^	476.2 ± 30.0 ^b^	2.6 ± 0.6 ^a^	2.9 ± 0.1 ^a^	1384.7 ± 135.0 ^d^	1194.4 ± 27.0 ^c^
Na	51.1 ± 1.8 ^a^	509.8 ± 15.0 ^b^	57.7 ± 1.2 ^a^	627.8 ± 1.3 ^c^	51.0 ± 1.0 ^a^	69.7 ± 1.9 ^a^	590.5 ± 11.0 ^c^	1859.9 ± 53.0 ^d^
Mg	40.1 ± 1.0 ^a^	80.2 ± 0.4 ^c,d^	46.3 ± 0.5 ^b^	101.0 ± 1.0 ^e^	77.7 ± 0.9 ^c^	81.4 ± 2.8 ^d^	330.3 ± 1.0 ^f^	302.4 ± 2.0 ^e^
Ca	10.8 ± 5.0 ^a,b^	74.6 ± 18.0 ^c^	28.7 ± 2.0 ^b^	81.3 ± 1.0 ^c^	0.0 ± 0.0 ^a^	17.5 ± 2.4 ^a,b^	254.6 ± 10.0 ^d^	270.7 ± 16.0 ^d^
Mn	0.29 ± 0.02 ^a^	1.47 ± 0.10 ^c^	0.64 ± 0.04 ^b^	2.07 ± 0.02 ^d^	0.26 ± 0.05 ^a^	0.54 ± 0.05 ^b^	7.19 ± 0.10 ^f^	6.53 ± 0.10 ^e^
Zn	1.92 ± 0.30 ^b^	1.40 ± 0.20 ^a^	2.41 ± 0.08 ^c^	2.69 ± 0.13 ^c^	12.19 ± 0.04 ^f^	28.66 ± 0.19 ^g^	5.63 ± 0.20 ^e^	5.01 ± 0.10 ^d^
Fe	1.07 ± 0.03 ^a^	2.52 ± 0.02 ^b^	2.96 ± 0.19 ^b^	5.34 ± 0.07 ^c^	8.84 ± 0.68 ^d^	9.86 ± 0.13 ^e^	12.32 ± 0.01 ^g^	11.55 ± 0.40 ^f^
Cu	0.42 ± 0.04 ^a^	1.48 ± 0.03 ^f^	0.56 ± 0.01 ^b,c^	0.68 ± 0.02 ^d^	1.05 ± 0.05 ^e^	3.45 ± 0.08 ^g^	0.58 ± 0.01 ^c^	0.48 ± 0.01 ^a,b^
Cd	<LOD	<LOD	<LOD	<LOD	<LOD	<LOD	<LOD	<LOD

* n = 2; SD—standard deviation; MW-AD—microwave-assisted acid digestion; different letters (a–g) within the same row indicate significant differences between concentrations of analyzed elements in gingerbread samples (one-way ANOVA and Duncan test, *p* < 0.05).

**Table 3:** Antioxidant capacity of uniced and iced gingerbread cookies.

Method	Antioxidant Capacity * ± SD (mmol TE/100 g)									
	DES1-Xyl:MalA		DES2-ChCl:MalA		DES3-ChCl:LacA		70% MeOH		70% EtOH	
	Uniced	Iced	Uniced	Iced	Uniced	Iced	Uniced	Iced	Uniced	Iced
ABTS	0.59 ± 0.02 ^a^	1.22 ± 0.06 ^c^	1.05 ± 0.03 ^b,c^	3.28 ± 0.06 ^d^	1.20 ± 0.21 ^c^	0.84 ± 0.22 ^b^	4.81 ± 0.23 ^e^	5.52 ± 0.17 ^f^	5.41 ± 0.17 ^f^	5.36 ± 0.11 ^f^
DPPH	<LOD	<LOD	<LOD	<LOD	0.71 ± 0.15 ^a^	0.75 ± 0.10 ^a^	1.87 ± 0.56 ^b^	1.77 ± 0.67 ^b^	2.09 ± 0.93 ^b^	1.78 ± 0.48 ^b^
CUPRAC	0.04 ± 0.00 ^a^	0.71 ± 0.03 ^b^	0.06 ± 0.00 ^a^	1.17 ± 0.03 ^c^	1.54 ± 0.54 ^c,d^	1.12 ± 0.52 ^c^	1.73 ± 0.01 ^d,e^	1.79 ± 0.03 ^d,e^	2.13 ± 0.03 ^e^	2.06 ± 0.03 ^e^

* n = 5; SD—standard deviation; LOD—detection limit; different letters (a–f) within the same row indicate significant differences between antioxidant capacity of the studied gingerbread extracts (one-way ANOVA and Duncan test, *p* < 0.05).

## Data Availability

The original contributions presented in the study are included in the article, further inquiries can be directed to the corresponding author.
